# Seroepidemiology of leptospirosis in dogs from rural and slum communities of Los Rios Region, Chile

**DOI:** 10.1186/s12917-015-0341-9

**Published:** 2015-02-12

**Authors:** Maud Lelu, Claudia Muñoz-Zanzi, Brooke Higgins, Renee Galloway

**Affiliations:** Division of Epidemiology and Community Health, School of Public Health, University of Minnesota, 1300 S. Second st. Suite 300, Minneapolis, MN 55454 USA; Bacterial Special Pathogens Branch, Centers for Disease Control United States, 1600 Clifton Road NE, Atlanta, GA 30333 USA; Institute of Pathology, Austral University of Chile, Campus Isla Teja, Valdivia, Chile

**Keywords:** Canine leptospirosis, *Leptospira*, Canicola serogroup, Zoonosis, Ecology epidemiology, Chile

## Abstract

**Background:**

Leptospirosis is a zoonotic disease of global importance and often neglected as a public health problem due to lack of awareness, under-diagnosis and under-reporting. Animals serve as a source of transmission through the shedding of *Leptospira* in their urine. Because of their proximity to humans, dogs may play a role in human infections. In order to assess and mitigate leptospirosis in dogs and the risk of transmission to humans it is important to understand the epidemiology of leptospirosis under natural conditions. This study aimed to characterize leptospirosis in owned dogs from three distinct community types. Blood, dog and household data were collected from 265 dogs in 190 households from 12 communities representing farms, rural villages, and urban slums in the Los Rios region, Chile. Serologic profiles with a 20-serovar microagglutination test panel were obtained. Binomial and multinomial logistic regression models were used to evaluate the associations between spatial, ecological, socio-economic variables and overall seropositivity as well as seropositivity to serogroup Canicola.

**Results:**

Results from 247 dogs with no history of vaccination were used. Overall seroprevalence was 25.1% (62/247) with significant differences by community type: 10.9% (9/82) in dogs from farms, 22.3% (21/94) from rural villages, and 45.1% (32/71) from urban slums (p <0.001). This trend by community type was also observed for dogs with evidence of seropositivity to the Canicola serogroup. Factors associated with seropositive dogs included dog density and precipitation two-weeks prior to sampling. Presence of *Leptospira* positive puddles collected from the peri-domestic household environment was also associated with increased seropositivity.

**Conclusions:**

Results suggest that leptospirosis is actively maintained in the dog population in this study region with notably distinct patterns by community type. Dog populations from rural villages, and urban slums in particular, showed evidence of high levels of transmission probably as a result of the combined effects of dog living conditions as well as community-level ecological and environmental factors.

**Electronic supplementary material:**

The online version of this article (doi:10.1186/s12917-015-0341-9) contains supplementary material, which is available to authorized users.

## Background

Leptospirosis is a water-related zoonotic disease of global health importance [1]. The disease is frequently not recognized and consequently severely neglected. The clinical course of human leptospirosis ranges in severity from asymptomatic or mild infection to severe illness including jaundice, renal failure, and hemorrhaging [2]. *Leptospira* bacteria are maintained in the environment through a complex transmission cycle in which humans and other mammals become infected after contact with urine from an infected host or with *Leptospira*-contaminated water or damp soil. Wild and domestic animals are reservoirs of pathogenic *Leptospira*; they maintain the leptospires in their proximal renal tubules in the kidney and shed the organism in the urine [1]. In order to assess, monitor, and mitigate the risk to humans it is necessary to understand the eco-epidemiology of *Leptospira* in the animal hosts.

Although rodents are often stated as the main source of human infections, dogs may also play an important role in transmission risk because of their proximity to humans. Dogs are recognized as hosts of *L. interrogans* serovar Canicola. Therefore, Canicola-infected dogs are disseminators of this serovar into the environment [3]. Usually *L. interrogans* serovar Canicola is the most frequent serovar found in infected dogs [4-6]; however, in areas where vaccination against serovars Canicola and *L. interrogans* serovar Icterohaemorrhagiae are common, other serovars are more prevalent, for example serovars *L. interrogans* serovar Autumnalis or *L. kirschneri* serovar Grippotyphosa [7-9]. Changing patterns of *Leptospira* infection in local stray dog populations has been reported by several studies [8,10-12]. Ward et al. [12] stated that such a change in the epidemiology of leptospirosis in dogs was likely influenced by new transmission patterns due to the evolving roles of wildlife and livestock in *Leptospira* transmission. However, knowledge and quantification of the major factors contributing to the new modes of transmission within communities remain unknown.

These and other knowledge gaps may also explain inconsistent results regarding the importance of risk factors in epidemiological studies of canine leptospirosis. Researchers often assess the role of the following dog/community characteristics: breed, age, location (urban/suburban vs. rural), gender, season, wildlife exposure, and past vaccination. Despite general trends, no conclusive factors have been identified as significant, ubiquitous risks for infection. Many authors agree that urban dogs have a higher risk of infection than rural dogs due to higher densities of dogs and rodents which increase exposure risk among susceptible animals [8,13]. Furthermore, dogs that live in peripheral urban areas where the sanitary conditions and infrastructure are precarious, compounded with biological and non-biological trash, open sewers and close proximity to other animal species constitute populations particularly at risk [14]. Thus, infection levels may also be higher in slum communities than in central urban or rural communities. This suggests ecologically different systems in which *Leptospira* spreads among dogs; however, studies focused on assessing the role of both ecological and socio-demographic or household variables on dog infections are limited.

Leptospirosis is endemic in Chile with human cases being reported sporadically [15] and abundant evidence of infection in livestock [16] and in rodents [17]. The limited local data available on canine leptospirosis revealed a prior estimate of sero-prevalence of 14.8% [6]. As part of a larger study on the eco-epidemiology of leptospirosis in Los Rios Region, the objective of this study was to characterize the serologic profiles in dogs from urban slums, rural villages, and farms and to attempt to investigate factors influencing distinct observed patterns.

## Methods

### Study population and data collected

The data used in this study are part of a larger study on the eco-epidemiology of leptospirosis that is being carried out in the Los Rios Region in the south-central part of Chile (Latitude: 39°15’S - 40°33’S, Longitude: 73°43’W-71°35’W) [17]. The region’s climate is characterized as temperate rainforest. Annual cumulative rainfall is 2,588 mm, however the central valley can get up to 1,200 mm per year, while the Andes Mountains receives 5,500 mm. Average temperature is 17°C in summer and 8°C in winter. Communities (four of each type) were selected based on the following definitions: i) Slums: informal settlements in the outskirts of a major city characterized by substandard housing, ii) Villages: rural community settlements away from major cities where households are clustered together, iii) Farms: dispersed households, typically small family farms, located in a specific rural locality. Communities were selected from areas where most settlements in the region are located; the central valley and near the region’s capital. Almost all communities were located at an altitude of 0-100 m, except for one village (C-2) and two farm communities (D-1 and D-3) which had an altitude of 100-200 m.

Between August 2010 and March 2012, 422 households participated in the study, with up to 40 households per community. Information on socio-demographic characteristics, living conditions, presence of domestic animals and livestock, evidence of rodents in the households were obtained via questionnaires. The dog density in each community was estimated by averaging the number of dogs per household multiplied by the total number of households in each community divided by the surface of the community, including a 250 meter buffer around households (see details in Additional file 1).

Measures of daily rainfall (mm) for the duration of the study and a 70-day buffer on each side were obtained from the National Aeronautics and Space Administration (NASA) Tropical Rainfall Measuring Mission (TRMM) dataset (Washington, D.C., United States) compiled with 3B42 algorithm, version 7. Each community was ascribed precipitation data based on its position within the 0.25 by 0.25 degree spatial grid used by NASA databases, resulting in 6 NASA dataset locations for the 8 communities. These data were downloaded using the MIRADOR search tool and were provided in 3-hour increments from which a daily measure of total precipitation was calculated. A similar approach was used to obtain surface temperature (K) from the Global Land Data Assimilation System (GLDAS) Noah model dataset version L4 (NASA, Washington, D.C., United States) compiled for the same duration of time. These measurements were also provided in 3-hour increments which were then averaged to calculate surface temperature estimates for the day. All owners gave written consent for their dogs to participate in the study. The study protocol was approved by the University of Minnesota’s Institutional Review Board (No. 0903 M62042) and Institutional Animal Care and Use Committee (No. 0904A63201) and the Austral University’s Human and Animal Ethics Committee (No. 01/09).

### Detection of *Leptospira*-specific antibodies

Sera of sampled dogs were tested for the presence of *Leptospira* antibodies using a microscopic agglutination test (MAT) including a panel of 20 serovars, representing 17 serogroups: *L. interrogans* serovars Australis, Bratislava, Autumnalis, Bataviae, Canicola, Djasiman, Grippotyphosa, Icterohaemorrhagiae, Mankarso, Pomona, Pyrogenes, and Wolffi, *L. borgpetersenii* serovars Ballum, Javanica, and Tarassovi, *L. kirschneri* serovar Cynopteri, *L. santarosai* serovars Borincana, Alexi, and Georgia, and *L. weilii* serovar Celledoni. MAT was carried out at the Centers for Disease Control, United States. Sera were diluted two-fold, starting at 1:100 dilution. The reported titer was the highest dilution of serum that agglutinated at least 50% of the cells for each serovar tested. Resulting agglutination titers were read using darkfield microscopy. Titers of 1:100 or higher were considered positive [18].

### Statistical analyses

#### Description of MAT titer distributions and seroprevalence

Dogs were classified as seropositive or seronegative to determine overall prevalence of past infection (and 95% confidence interval), by community and by community type. Descriptive frequencies were compared using chi-square tests. Serological profiles or distribution of titers against each reference strain in the MAT panel were described by community type using frequencies and were visually displayed using heat maps. For the heat maps, a negative titer (no agglutination at 1:100) was given the value of 50 and titers were plotted as log_2_(reciprocal titer/50). Dogs reported as previously vaccinated for leptospirosis by the head of household and dogs with missing vaccination history were excluded from all analyses.

#### Ecological and demographic variables examined in the analysis of infection risk

Because dogs were sampled at known geographic locations (represented by the latitude and longitude of their household), the Moran index method from the R package ade4 [19] was used to test for spatial autocorrelation. Possible spatial autocorrelation of positive dogs was detected only for community C-3 where 11 of 31 dogs were positive (p = 0.04). Variables were obtained from survey questionnaires, computation of spatial variables, and other studies that are part of the same larger project [17]. The following variables were considered most relevant: community type (farms, villages, slums), prior leptospirosis vaccination (0: no, 1: yes), head of household finished high school (0: no, 1: yes), number of persons in the household, income category (0: < USD $328/month, 1: ≥USD $328/month), type of waste disposal (0: no waste disposal, 1: sewerage or septic tank, 2:latrine), type of trash removal (0: by other means, 1: by truck), flow accumulation index derived from elevation (weighted sum of how many cells contribute to the water that flows into each cell of 90 m x 90 m), number of households within 100 m of each dog household, number of dogs in the household, dog density per community, sex (0: female, 1:male), type of dog (0: companion, 1:guardian), number of livestock per household, and number of rodents trapped per household. Presence of water samples in the study households positive for pathogenic *Leptospira* by PCR was obtained as reported previously (0: no PCR positive samples, 1: ≥1 PCR positive sample) [20]. Weather variables included 7-day cumulative precipitation two weeks and one month prior to sampling, cumulative precipitation during the 30 days prior to sampling and average precipitation during the 30 days prior to sampling. Variables for temperature were calculated in the same manner. In order to assess the potential correlations between variables, a mixed multivariate descriptive analysis combining quantitative and qualitative variables [21] was performed.

#### Analysis of factors associated with overall *Leptospira* seropositivity and with presumed seropositivity to the Canicola serogroup

A binomial logistic regression with random effects for household and community was performed on the outcome defined as “MAT positive” or “MAT negative”. Additionally, a multinomial logistic regression (R package nnet) [22] was performed using the same explanatory variables on a 3-level multinomial outcome which was defined as “Negative”, “Presumed seropositive to Canicola serogroup”, and “Seropositive to other serogroup”. Acknowledging the limitations of MAT to identify the specific serovar responsible for a given infection [23-25], for the purpose of this analysis, the presumptive infecting serogroup was established based on the serogroup with a titer at least two dilutions higher than any other titer in the panel. Seropositive dogs that did not meet this definition for Canicola seropositivity were classified as “Seropositive to other serogroup”. For both regression models, model selection was based on statistical significance, comparison of corrected Akaike information criterion values (AICc), and examination of model assumptions, while considering the need to adjust the model for potential confounders. Model selection also examined biologically plausible interactions between two variables. Effect of the significant variables was reported as odds ratio (OR) and corresponding 95% confidence intervals following standard methods.

## Results

**Figure 1:**
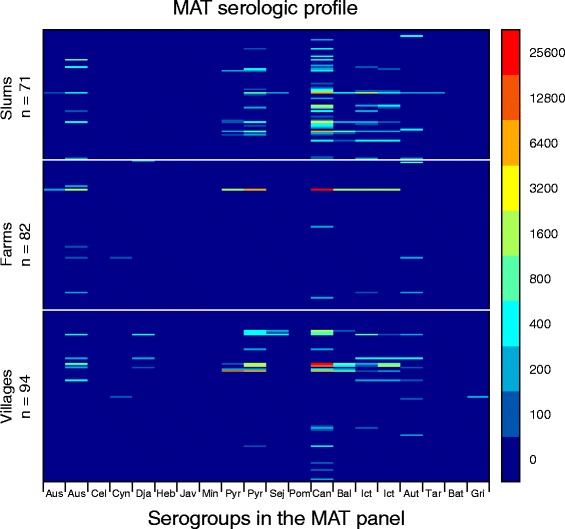
**Heat map displaying leptospirosis MAT serologic profiles for dogs in slums, villages, and farms.** Individual titers for each serogroup in the MAT panel are shown for all 247 dogs in the study and color coded accordingly.

From the 422 households that answered the questionnaire, 353 had at least one dog with a total of 699 dogs reported. The number of dogs per household ranged from 0 to 10, with an average number of dogs per household of 2.2 (sd = 1.34), 1.6 (sd = 1.59) and 1.1 (sd = 0.92) in farm, village and slum communities, respectively (p < 0.001). Blood was collected from 265 dogs from 190 households. Sixteen dogs that had past vaccination against *Leptospira* and two that did not have vaccination status were excluded from all analyses*.* A total of 62 dogs out of 247 (25.1%, 95% CI: 19.8 - 31.0%) had reciprocal titers of 100 or higher for one or more *Leptospira* serovars represented in the MAT panel. Seroprevalences significantly differed by community type (p < 0.001), with 10.9% (95% CI: 5.1 – 19.8%), 22.3% (95% CI: 14.4 – 32.1%) and 45.1% (95% CI: 33.2 – 57.3%) for the farm, village and slum communities, respectively (Table 1). From the panel of 20 serovars, titers to 11 serogroups (14 serovars) were detected: Australis (serovars Australis and Bratislava), Autumnalis, Ballum, Canicola, Cynopteri, Djasiman, Grippotyphosa, Icterohaemorrhagiae (serovars Icterohaemorrhagiae and Mankarso), Pyrogenes (serovars Alexi and Pyrogenes), Sejroe (serovar Wolffi) and Tarassovi. Display of the serologic profiles (titer to each serovar in the MAT panel for each seropositive dog) showed that titers ranged from negative to 1:25,600. MAT serologic profiles showed the dominance of titers to the Canicola serogroup across community types but with higher titers in dogs from slums and rural villages than from farms as well as titers to a broader range of MAT panel serogroups in dogs from villages and farms than in dogs from slums (Figure 1).

**Table 1:** **Seroprevalence of leptospirosis in dogs from slum, village, and farm communities from Los Rios Region, Chile**

	**Community**	**Dogs with titer ≥ 1:800**	**Seroprevalence (any titer)**		
Slums	U-1	1/6 (16.7%)	6/16 (37.5%)	32/71 (45.1%)	62/247 (25.1%)
	U-2	5/16 (31.3%)	16/24 (66.7%)		
	U-3	0/5 (0%)	5/14 (35.7%)		
	U-4	1/5 (20%)	5/17 (29.4%)		
Villages	C-1	0/8 (0%)	8/31 (25.8%)	21/94 (22.3%)	
	C-2	0/2 (0%)	2/23 (8.7%)		
	C-3	6/11 (54.5%)	11/29 (37.9%)		
	C-4	--	0/11 (0%)		
Farms	D-1	--	0/6 (0%)	9/82 (10.9%)	
	D-2	0/4 (0%)	4/30 (13.3%)		
	D-3	0/1 (0%)	1/20 (5%)		
	D-4	2/4 (50%)	4/26 (15.4%)

**Figure 2:**
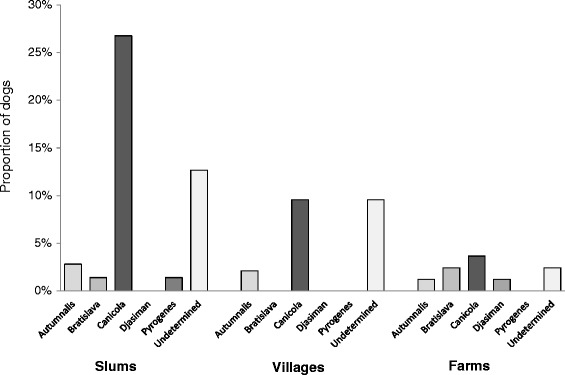
**Distribution of the presumed infecting serogroup among 247 non-vaccinated dogs enrolled in three community types (Farms, Villages and Slums) from Los Rios Region, Chile.** For each seropositive dog, the infecting serogroup was defined as the serogroup with a titer at least two dilutions higher than any other titer in the dog’s profile. Undetermined corresponded to dogs for which this definition could not be applied.

Fifty percent of the seropositive dogs (31/62), and 12.6% (31/247) of all dogs, were classified as seropositive for the Canicola serogroup. Among those dogs, titers to Canicola ranged from 1:100 to 1:25,600 and 10 had titer ≥1:800. Sixteen of the 31 (51.6%) dogs had titers to serogroup Canicola only, ranging from 1:100 to 1:400. The proportion of dogs with Canicola seropositivity increased from farms (3/82, 3.7%), to villages (9/94, 9.6%), and was highest in slums (19/71, 26.8%) (p < 0.001) (Figure 2).

### Associations among household and ecological variables examined

The first and second axes of the mixed multivariate analysis respectively accounted for 17.3% and 10.8% of the variations among the variables. These two axes adequately separated the three types of community. The analysis indicated that slums were associated with high number of households within 100 m., low number of livestock, low income and no waste disposal and high dog density. Villages were associated with more heads of household who finished high school, use of sewer system or septic tank and high dog density. Farms were associated with high number of livestock, low number of households within 100 m., use of latrine, and removal of trash other than by truck. Thus these factors differentiated the three types of community (See Additional files 2 and 3 for details).

#### Variables associated with overall *Leptospira* seropositivity

Model selection resulted in a final model that included community type and 7-day cumulative precipitation two weeks prior to sampling, while adjusting for dog density. Dog density showed to confound some of the effect of community type as seen by a 20% decrease in the OR in the adjusted model. Odds of seropositivity were significantly higher in dogs from urban communities compared with farms (p <0.001, OR = 8.06, 95% CI: 2.21-29.42) but there was no difference between dogs from rural villages and farms (p = 0.105). Although not significant at p = 0.05, increase in rainfall two weeks prior to sampling was associated with an increase in seropositivity (p = 0.08, OR = 1.20 for a 10 mm increase in rainfall, 95% CI: 0.98-1.46). Furthermore, the observed data suggested a positive association between dog density and seroprevalence in each community type and in the urban slums in particular (Additional file 4). Analysis of a subset of 132 dogs from 95 households from all 12 communities where puddle samples were collected and tested for *Leptospira* by PCR revealed community type effects consistent with the overall model described above, as well as an association between dog seropositivity and presence of PCR positive puddles in the household (p = 0.014, OR = 3.87, 95% CI: 1.32-11.35).

#### Variables associated with positivity to Canicola serogroup

The final multinomial model identified dog density as significantly associated with seropositivity to the Canicola serogroup (p = 0.030) compared with dogs with no evidence of past infection (seronegative). An interaction between community type and cumulative precipitation two weeks prior to sampling showed that increased rainfall was strongly associated with seropositity to Canicola serogroup compared with seronegative dogs, although this was only identified in rural villages (p = 0.078). An increase in precipitation by 1 mm was associated with a 6% increase in the odds Canicola seropositivity. No variables were significantly associated with non-Canicola seropositivity.

## Discussion

We presented a study investigating the potential interplay between biological, ecological, spatial and socio-demographic factors on exposure to *Leptospira* in dog populations from a temperate region of southern Chile. The estimated seroprevalence reported here corresponds to unvaccinated, apparently healthy dogs selected based on enrollment of households into the study. Vaccination status was ascertained based on the head of household’s information which may be subject to error; however, this impact is likely to be minimal as preventive veterinary care is rarely done in the low-income populations represented in this study. The overall seroprevalence of 25.1% (95% CI: 19.8 - 31.0%), was similar to the estimates reported in other studies. Bastista et al. [26] found a seroprevalence of 21.4% in a study population of 285 stray dogs in Paraíba, Brazil. Aguiar et al. [27] also reported an overall seroprevalence of 27.3% (90/329) in Brazil, with no statistical differences between urban and rural communities. In the city of Temuco, Chile, seroprevalence in stray dogs was estimated to be 21.3% [28]. However, direct comparisons of seroprevalences are often difficult as the detection method, the MAT panel of serovars and the vaccination history of dogs vary among studies. The selection method may greatly impact comparability. For example, a previous study reported a seroprevalence of 14.8% in dogs from the city of Valdivia [6], which is lower than the prevalence in dogs from the two slum study communities located in the same city (35.3% in U-1 and 66.7% in U-2). This difference could be attributed to differences inherent to slum communities and to difference in enrollment methods as the Riedemann’s study used dogs brought into veterinary clinics, while this study enrolled dogs directly from households in the communities.

Use of MAT titer results to infer the infecting serovar has shown inconsistencies; however, it can give an indication of the serogroups present in a population [23]. Based on the definition of presumptive infecting serogroup applied here, a titer that is at least two dilutions higher than titers for other serogroups, suggested that serologic evidence of infection by serogroup Canicola was significant overall (50% of seropositive dogs) and in each community type. Moreover, slightly more than half of those dogs had titers to Canicola only which indicated more specific reactions. The predominance of titers to Canicola was also found in a study from Valdivia, Chile [6], Sao Paulo, Brazil [4], and South Africa [5]. These findings tend to suggest that infection by Canicola serogroup is important in the study population and, more importantly, examination of the entire MAT serologic profiles reveals distinct patterns by community type. Dogs from urban slums tended to show titers to a limited number of MAT serogroups and dogs from rural villages and farms to a much wider range of serogroups in the panel. Additionally, titers to the Canicola serogroup were much higher in farms and rural villages than in slums (Figure 1). Although we can only speculate, these patterns seem to be indicative of the broader range of *Leptospira* exposures by dogs in rural areas as a result of a broader diversity of potential hosts that could be transmitting *Leptospira* to dogs. The observed association between dog seropositivity and presence of a *Leptospira* contaminated peri-domestic environment could denote shedding from infected dogs as well as from other potential hosts sharing that environment. Further studies aim at clarifying the role of this shared environment on transmission of leptospirosis within communities are needed. Molecular analysis of the environmental study samples is being carried out.

Some correlations between titers to some MAT serovars, notably Canicola, Pyrogenes and Ballum were observed. A similar correlation between titers to serovars Pyrogenes and Canicola was reported in dogs from South Africa [5]. In their study the authors suggested that dogs may have had exposures to both serovars. Here, as some titers not only were often positive for both serovars but also increased together, it is more likely that this association is caused by cross-reaction than co-infection. Among seropositive dogs, 30.6% reacted to the serovar Icterohaemorrhagiae strain in the panel; however, Icterohaemorrhagiae was never found as the highest titer in a single sample. Considering the presence of infected rodents [17] and of livestock in farms and some household villages, infection of dogs from other hosts is certainly possible. We are not able to establish those associations beyond epidemiological evidence; but other studies have documented infection in dogs with isolates from non-Canicola serogroups including Hebdomadis [29] and Icterohaemorrhagiae [30] among others.

Host density is a known important parameter in infectious disease transmission [31]. Results consistently identified dog density as associated with increased seropositivity, overall and for the Canicola serogroup. Urban slums had the highest seroprevalence which, in the model, was partly explained by the high dog density. In highly populated slums in Brazil, where high density of dogs is expected, street access was one of the most important risk factors for infection [32,33]. These studies support that in high density communities, such as slums or even villages with large number of dogs, the risk of infection may be increased because of the dog’s ability to roam freely within a limited space. If serovar Canicola strains are well adapted to be maintained in the dog population, high density may produce more dog-to-dog contacts, more transmission, and increased amount of *Leptospira* shed in the environment, which in turn may favor indirect transmission.

Increased precipitations may improve the survival of leptospires in the environment and it is often reported as a risk factor for infection [33,34]. Although statistically significant at p < 0.1, it is noteworthy that among the various variables examined for the effect of precipitation, increased precipitation two weeks prior to sampling was consistently found to be associated with *Leptospira* seropositivity. Single samples and assessment of immunological responses only are limitations of the study but the 2-week cutoff was chosen to provide enough time for dogs to develop an immune response if precipitation prior to collection was indeed a predictor of infection. The found association may be driven by the number of dogs with MAT titer ≥1:800 which are suggestive of increased transmission, and therefore, a better explanatory variable for more recent infections rather than past infections in not vaccinated dogs [35,36]. This is also consistent with the finding of increased rainfall and Canicola seropositivity but in dogs from villages only, where a large number of dogs had high titers (Figure 1, Table 1).

The high sero-prevalence in dogs observed in this study, including evidence of active transmission, may pose a public health problem to humans. Several studies assessing the risk factors of human *Leptospira* infection found that the presence of dogs in the household increased the risk of human infection [37-40]. For example, in Buenos Aires, in the region with the highest prevalence of leptospirosis in humans, 41% of the human patients were found to be infected with serovar Canicola, suggesting direct or indirect dog to human transmission [41]. Furthermore, having a high seroprevalence of leptospirosis in dogs also puts children at risk. In Texas, dogs from households of several children diagnosed with leptospirosis were found to be infected with serovar Canicola [42]. The authors suggested that the disease transmission may have occurred when both children and dogs played in the pools of water caused by heavy rains. In this study site, pathogenicity of the local circulating strains is unknown and clinical diagnosis is greatly underestimated [15]; however, past serosurveys showed ample evidence of opportunity for human infection [42]. Investigation of infection in people in the study communities is in progress.

## Conclusions

We highlighted the need to understand the ecology of *Leptospira* in the various animal hosts and to elucidate the specific drivers of the community-level differences observed. The use of population-level enrollment and complete serologic profiles from a large MAT panel may contribute additional information on patterns among titers which, if examined repeatedly over time, may help monitor changes in local transmission dynamics and serosurveillance.

## Additional files

Additional file 1:
**Examples of aerial photographs of each community type (urban slums, villages, and farms) with a 250 m buffer around the households.** These images were used to estimate the surface of each community enrolled in the study. Additional file 2:
**Factorial map of the Hill and Smith principal component analysis of the household and ecological variables examined in the study: first (x-axis) and second (y-axis) (** ***a*** **), considering the quantitative variables only (** ***b*** **).**
Additional file 3:
**Factorial map of the Hill and Smith principal component analysis of the study variables.** Analysis was done grouping the individuals by modalities for each categorical variable. Additional file 4:
**Display of observed seroprevalence and dog density (dogs/km** ^**2**^ **) for each community type.** Data showed consistently a positive trend with the exception of one village community (C-3) which showed low density and high prevalence. This community showed spatial autocorrelation of positive cases, high reciprocal MAT titers, as well as higher rainfall than the other villages.
