# Supplementary figures and images for: Seroepidemiology of leptospirosis in dogs from rural and slum communities of Los Rios Region, Chile
Source: BMC Vet Res. 2015 Feb 12;11:31. doi: 10.1186/s12917-015-0341-9 (PMC4329218; doi:10.1186/s12917-015-0341-9)

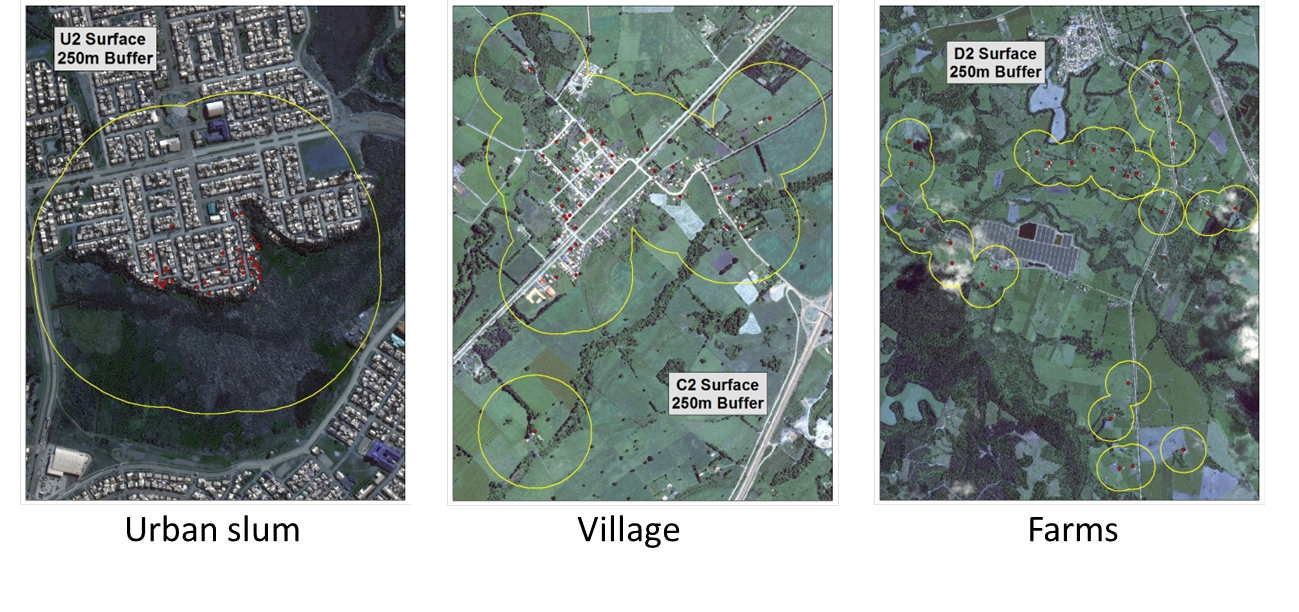

Supplement: Additional file 1: — Examples of aerial photographs of each community type (urban slums, villages, and farms) with a 250 m buffer around the households. These images were used to estimate the surface of each community enrolled in the study. [file 12917_2015_341_MOESM1_ESM.docx]

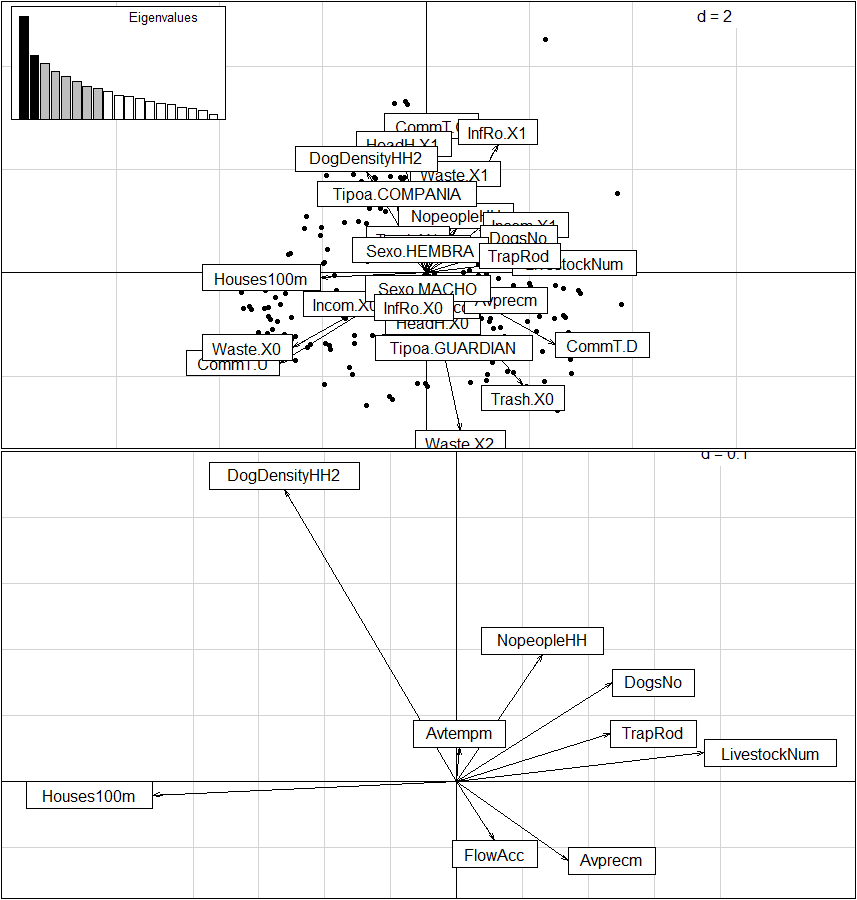

Supplement: Additional file 2: — Factorial map of the Hill and Smith principal component analysis of the household and ecological variables examined in the study: first (x-axis) and second (y-axis) ( a ), considering the quantitative variables only ( b ). [file 12917_2015_341_MOESM2_ESM.docx]

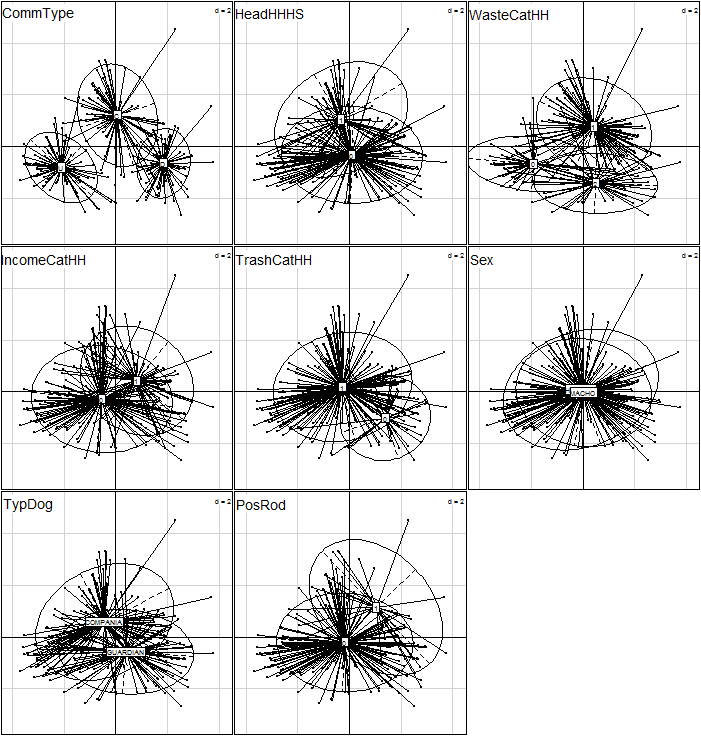

Supplement: Additional file 3: — Factorial map of the Hill and Smith principal component analysis of the study variables. Analysis was done grouping the individuals by modalities for each categorical variable. [file 12917_2015_341_MOESM3_ESM.docx]

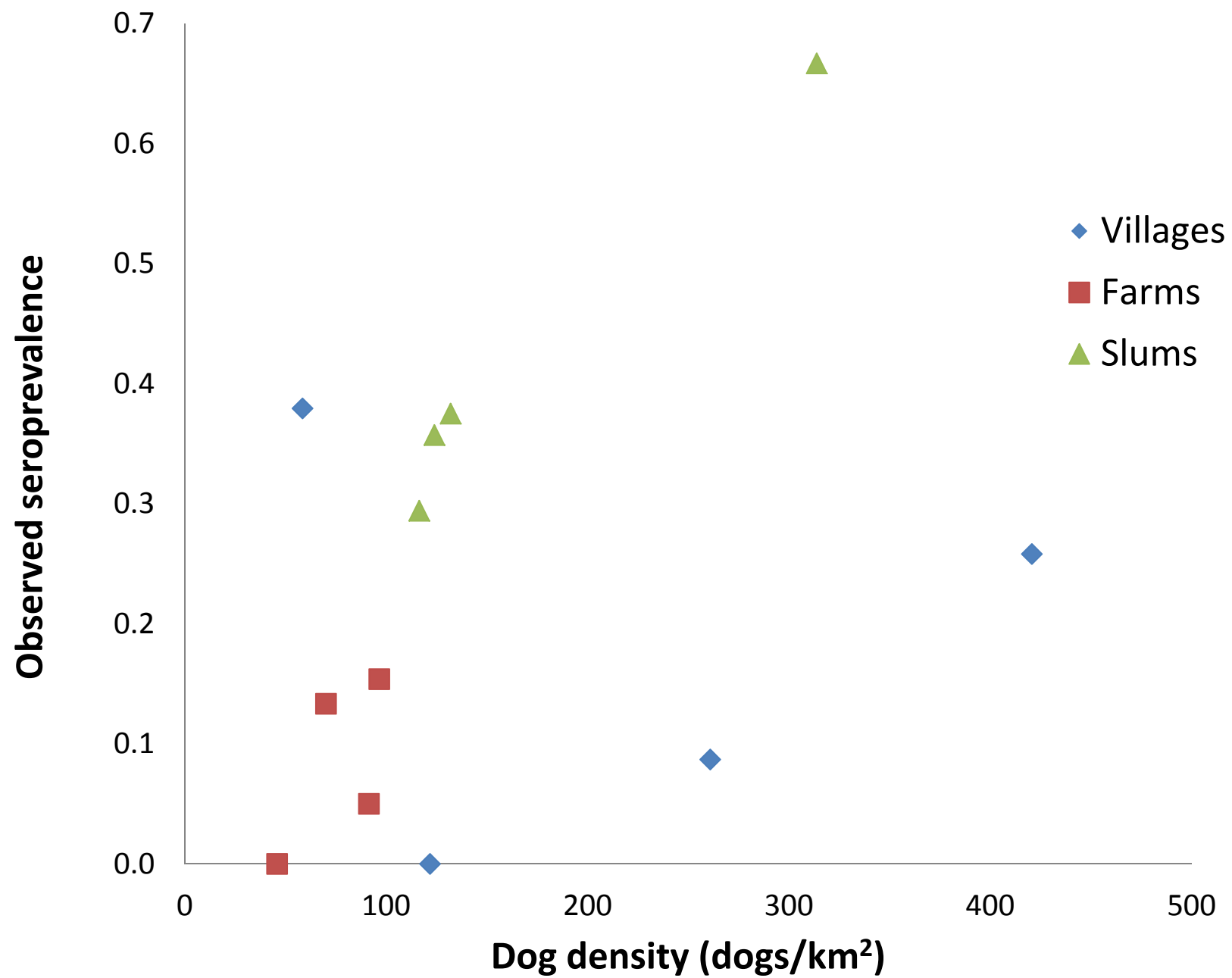

Supplement: Additional file 4: — Display of observed seroprevalence and dog density (dogs/km 2 ) for each community type. Data showed consistently a positive trend with the exception of one village community (C-3) which showed low density and high prevalence. This community showed spatial autocorrelation of positive cases, high reciprocal MAT titers, as well as higher rainfall than the other villages. [file 12917_2015_341_MOESM4_ESM.pdf]
